# Application of geopolymers synthesized from incinerated municipal solid waste ashes for the removal of cationic dye from water

**DOI:** 10.1371/journal.pone.0239095

**Published:** 2020-11-05

**Authors:** Mohammad A. Al-Ghouti, Mariam Khan, Mustafa S. Nasser, Khalid Al Saad, OON Ee Heng

**Affiliations:** 1 Department of Biological and Environmental Sciences, College of Arts and Sciences, Qatar University, Doha, State of Qatar; 2 Gas Processing Center, College of Engineering, Qatar University, Doha, State of Qatar; 3 Department of Chemistry and Earth Sciences, College of Arts and Sciences, Qatar University, Doha, State of Qatar; 4 Domestic Solid Waste Management Centre (DSWMC), Doha, State of Qatar; Indian Institute of Technology Patna, INDIA

## Abstract

In this study, municipal solid waste bottom ash (MSW-BA) and fly ash (MSW-FA) were used as a source of aluminosilicate to prepare geopolymer (GEO) adsorbents (GEO-MSWBA and GEO-MSWFA) for the removal of methylene blue (MB) from water. The effects of temperature, pH, and initial concentration on the MB adsorption onto GEO-MSWBA and GEO-MSWFA were evaluated. The adsorption isotherms parameters and thermodynamics were also determined. Detailed physical and chemical characterizations of the prepared adsorbents were carried out to further understand their impact on MB adsorption. The results from the scanning electron microscopy revealed a uniform granule-sphere like structure on both prepared geopolymers, which would facilitate the MB adsorption onto the adsorbents. The X-ray diffraction allowed observation of the microstructural transformations that occur after the alkaline activation. The surface areas of the GEO-MSWBA and the GEO-MSWFA were recorded as 32.78 m^2^/g and 4.5 m^2^/g, respectively. From the Fourier transform infrared, a stretching vibration of the aluminosilicate tetrahedral was observed, which indicated the success of geopolymerization. The prepared geopolymers showed a high capability of MB adsorption from an aqueous solution. The adsorption process was best suited and explained using the Langmuir isotherm model with a maximum adsorption capacity of 666.7 mg/g for the GEO-MSWBA (at 25°C) and 769.2 mg/g for the GEO-MSWFA (at 35°C). The positive value of the enthalpy (ΔH^*o*^) for the GEO-MSWBA suggested the reaction favored endothermic reaction while the negative value of entropy (Δ*S*^*o*^) indicated a solid/liquid random interaction. On the other hand, the negative ΔH^*o*^ value for the GEO-MSWFA indicated the reaction followed an exothermic reaction causing energy to be released, the positive Δ*S*^*o*^ value indicated a good affinity at the solid-liquid surface. The overall negative value for Gibbs free energy (Δ*G*^*o*^) for both adsorbents suggested the adsorption was spontaneous and feasible. It was also inferred that *n*- *π* interaction, direct and indirect hydrogen bond, and electrostatic interaction between the MB and the prepared geopolymers facilitated the adsorption process. The current study shows that the GEO-MSWBA and the GEO-MSWFA have a great potential of removing MB as a cationic dye from water without performing any sort of laborious pretreatments.

## 1. Introduction

The treatment of major water pollutants such as toxic metals, drugs, and dyes has become one of the major environmental concerns in the scientific community [1, 2]. Various industries including textiles, plastics, and cosmetics, release huge amounts of dyes into the water streams on a daily basis. Dyes that are released into the environment can indeed reduce water quality by reducing water clarity and aesthetic value. It may also influence photosynthetic activities, which can pose several health risks for aquatic organisms [3, 4]. Furthermore, prolonged exposure to these dyes can have severe consequences for human health and ecosystem due to their mutagenic, toxic, and carcinogenic properties, which can pose lethal effects [5, 6].

Basic dyes are one of the common types of dyes that are generally used around the world including methylene blue (MB). Various studies have highlighted that overtime exposure to MB can cause severe health impact on humans, such as tissue necrosis, jaundice, and vomiting [7]. There are various treatments present that are widely utilized for the removal of dyes from water, including biological treatment, chemical degradation, and physical adsorption. Biological treatments are commonly used technique have reported to not yield satisfactory color elimination because of various factors, including the complex structure and artificial origin [8]. Therefore, further treatment is often required to remove dyes efficiently [9, 10]. Owing to its simplicity, cost, and high efficiency, adsorption is by far the most widely used method for the removal of dyes. It is claimed, that adsorption may produce high quality treated effluent without secondary harmful substances [4]. Therefore, in the last few decades, low-cost adsorbent has been one of the main scope of scientific research. Various inexpensive and effective alternative adsorbents have been explored such as bio-sorbent, waste material from agriculture or industry, and synthesized materials have been studied successfully removal such cationic dye from aqueous solution [11–17].

Recent studies have highlighted the potential use of geopolymer (GEO) as adsorbent materials due to their rapid strength, environmental friendliness, ion exchange capacity and effectively solidifying toxic waste [18]. GEO is an inorganic polymer that can be prepared through a process called geopolymerization reaction, which can be classified as an eco-friendly reaction. It involves the dissolution of aluminum silicate with geological origin substance or industrial waste such as slag, ash, biomass, or red mud in a highly alkaline medium releasing Si and Al species [19]. Numerous studies have elaborated on the geopolymeric formation by the utilization of incinerated municipal solid waste (MSW) ash, namely municipal solid waste bottom ash (MSW-BA) and fly ash (MSW-FA) as a source of solid aluminosilicate [20–25]. The composition of the geopolymers consists of a three-dimensional network of free AlO_4_ and SiO_4_ tetrahedral, which are formed due to the breakdown of Si-O-Si and Si-O-Al bonds. These AlO_4_ and SiO_4_ are connected by oxygen corners with a typical 1:3 ratio of Si/Al, which are dissolved in an alkaline medium [26]. GEOs can be regarded as solid and stable aluminum-silicate materials. The most widely used alkali activators include sodium hydroxide, water glass (sodium silicate), potassium hydroxide, or a combination of these solutions in varying molarity. The utilization of GEOs as adsorbents has emerged as a promising concept for the removal of toxic substances from industrial and household effluents. It has also gained popularity for water de-colorization [27]. Recent studies have highlighted their potential to be used as suitable adsorbents for metal removal from aqueous solutions [27–31]. GEOs consist of highly interconnected and porous structure, high cation exchange capacity, and large affinity for metals, which facilitates in attracting cationic substance [18]. GEOs are mostly well known for their environmentally friendly properties. They are currently being used as a substitute for Portland cements (OPC) in various countries [6]. Recently, various researches showed great capacity of fly ash (FA) based adsorbent for the removal of methylene blue dye, and metals such as Cu^2+^, Cr^3+^, Cd^2+^and Pb^2+^ [17, 32, 33]. Yet very few articles have investigated the utilization of geopolymers based on MSW on the removal of dyes from aqueous media [25, 34–42].

This paper reports on the application of geopolymers synthesized from incinerated municipal solid waste ashes (GEO-MSWBA and GEO-MSWFA) on the removal of MB from water. The effects of temperature, pH, and initial MB concentration on the MB adsorption onto the GEO-MSWBA and GEO-MSWFA were evaluated. The adsorption isotherms parameters and thermodynamics were also determined. Detailed physical and chemical characterizations of the prepared geopolymers were carried out to further understand their impact on MB adsorption using Fourier transform infrared (FTIR), scanning electron microscopes-energy-dispersive X-ray spectroscopy (SEM-EDX) and X-ray diffractometry (XRD). Four different adsorption isotherm models, namely Langmuir, Freundlich, Temkin, and Dubinin-Radushkevich were studied in order to determine the mechanisms and efficiency of the adsorption process.

## 2. Materials and method

### 2.1. Reagent

Hydrochloric acid (HCl, 37%) and hydroxide (NaOH) extra pure pellets were obtained from Scharlu Barcelona (Spain). Sodium metasilicate was obtained from Sigma-Aldrich from (Germany). Extraction solutions were prepared with deionized water obtained from a Milli-Q system, 0.20 μm resistivity (Millipore, France).

### 2.2. Sample collection and preparation

To obtain a homogenous and representative sample, replicate samples were collected at different periods. MSW-BA and MSW-FA were freshly collected in a 5 kg representative sample from a local incinerator (Qatar Company: Domestic Solid Waste Management Centre (DSWMC)–Doha, State of Qatar). The permission of the sample collection was already granted by the Company. We confirm that the field studies did not involve endangered or protected species. To minimize the change in the physiochemical characteristics due to natural weathering, the ashes were immediately dried at 100 ^o^C for 24 h, ground at different particle sizes, and sieved through a standard sieve. Then the samples were kept in clean and isolated glass bottles. A particle size of less than 250 μm was used for the geopolymer preparation. Sodium silicate solution (Na_2_SiO_3_) was prepared by dissolving Na_2_SiO_3_ in water and heated in an electric stove at 175°C.

### 2.3. Preparation of the geopolymers

Geopolymer adsorbents were prepared by adding 30 g of the MSW-BA or MSW-FA to 8 mL of 5 M NaOH. The mixture was mixed for 10 minutes at room temperature to allow ions leaching and formation of a slurry mixture. Na_2_SiO_3_ solution was then added to the slurry specimen and stirred until a homogenous solution was obtained. The resulting paste was placed in an oven at 65°C for 72 h until the mixture was completely dry. Excess NaOH was removed from the geopolymer mixture paste by continuously washing with deionized water. The specimens were then crushed and sieved to obtain uniform smaller particles for the experiment. A particle size of less than 250 μm was used to perform the geopolymer characterizations and the adsorption studies. The mixed proportion of the geopolymer specimens were 60% ash (MSW-BA or MSW-FA), 16% NaOH, and 24% Na_2_SiO_3_ by weight [43]. The prepared geopolymers were named as GEO-MSWBA and GEO-MSWFA, respectively.

### 2.4. Characterization studies

The main functional groups of the prepared geopolymers were characterized by FTIR (PerkinElmer 400 Spectrum instrument using Universal Attenuated Total Reflectance, USA). FTIR analysis was carried out in the range of 4000–400 cm^−1^. The crystalline phase of the prepared geopolymers was analyzed via XRD (PANalytical Empyrean/Netherland). The morphology and microstructure of samples were visualized and analyzed by SEM (Nova™ Nano SEM 50 Series, FEI, USA). The pore size distribution and surface area were obtained using the Brunauer-Emmett-Teller (BET) analyzer (Quantachrome Corporation, Nova 3000).

### 2.5. Statistical analysis

Two way ANOVA was performed using Excel 2018 for the effect of pH, temperature, and the initial concentration on the MB adsorption process onto GEO-MSWBA and GEO-MSWFA.

### 2.6. Adsorption isotherm studies

All experiments were performed using the batch process. Four linearized forms of the isotherm models were used, namely Langmuir, Freundlich, Dubinin-Radushkevish, and Temkin to evaluate the adsorption capacity. A fixed amount of the geopolymer GEO-MSWBA or GEO-MSWFA (0.05 g) was added to a 50 mL of the MB solution at different pH values, temperatures, and initial MB concentrations, and placed in a temperature-controlled shaker at 130 rpm (shaking Incubator, MODEL: SSI10R-2, Orbital-Shaking). To ensure quality, control blank and two trials were prepared. Each solution concentration was measured under 663 nm [44] using a visible spectrophotometer (PerkinElmer Lamda 25UV/VIS spectrophotometer). The removal efficiency was calculated using Eq 1:

$$\mathrm{Removal}\;\mathrm{percentage}\;(\%)=\frac{C_{0}-C_{eq}}{C_{0}}x\;100$$ (1)

Where, C_0_ and C_eq_ are the initial and equilibrium of MB concentrations (mg/L), respectively.

The adsorption capacity was calculated using Eq 2:

$$\mathrm{Adsorption}\;\mathrm{capacity}\;\left(q_{e}\right)=\frac{\left(C_{0}-C_{eq}\right)\times V}{m}$$ (2)

Where q_e_ is the amount of MB adsorbed by the geopolymer (mg/g), V is the volume (L), and m is the mass of the prepared geopolymer (g).

#### 2.6.1 Effect of pH, initial concentration, and solution temperature

A 0.05 g of the prepared geopolymer was shaken with a 50 mL of the 1000 mg MB /L solution at 25 ^o^C for 48 h at various pH values (2.0, 4.0, 6.0, 8.0, and 10.0). The pH values were adjusted using a pH meter (Jenway 370). 0.1 M sodium hydroxide (NaOH) and 0.1 M hydrochloric acid (HCl) were used to adjust the pH value. Different initial MB concentrations were studied (100, 200, 300, 400, 500, 600, 800, 900, and 1000 mg/L). The MB adsorption was also studied at various temperatures (25°C, 35°C, and 45°C). The removal efficiency and adsorption capacity were measured after the system reached equilibrium, which was 48 h.

## 3. Results and discussion

### 3.1. Characterization of the prepared Geopolymers

#### 3.1.1 SEM and XRD

**Fig 1:**
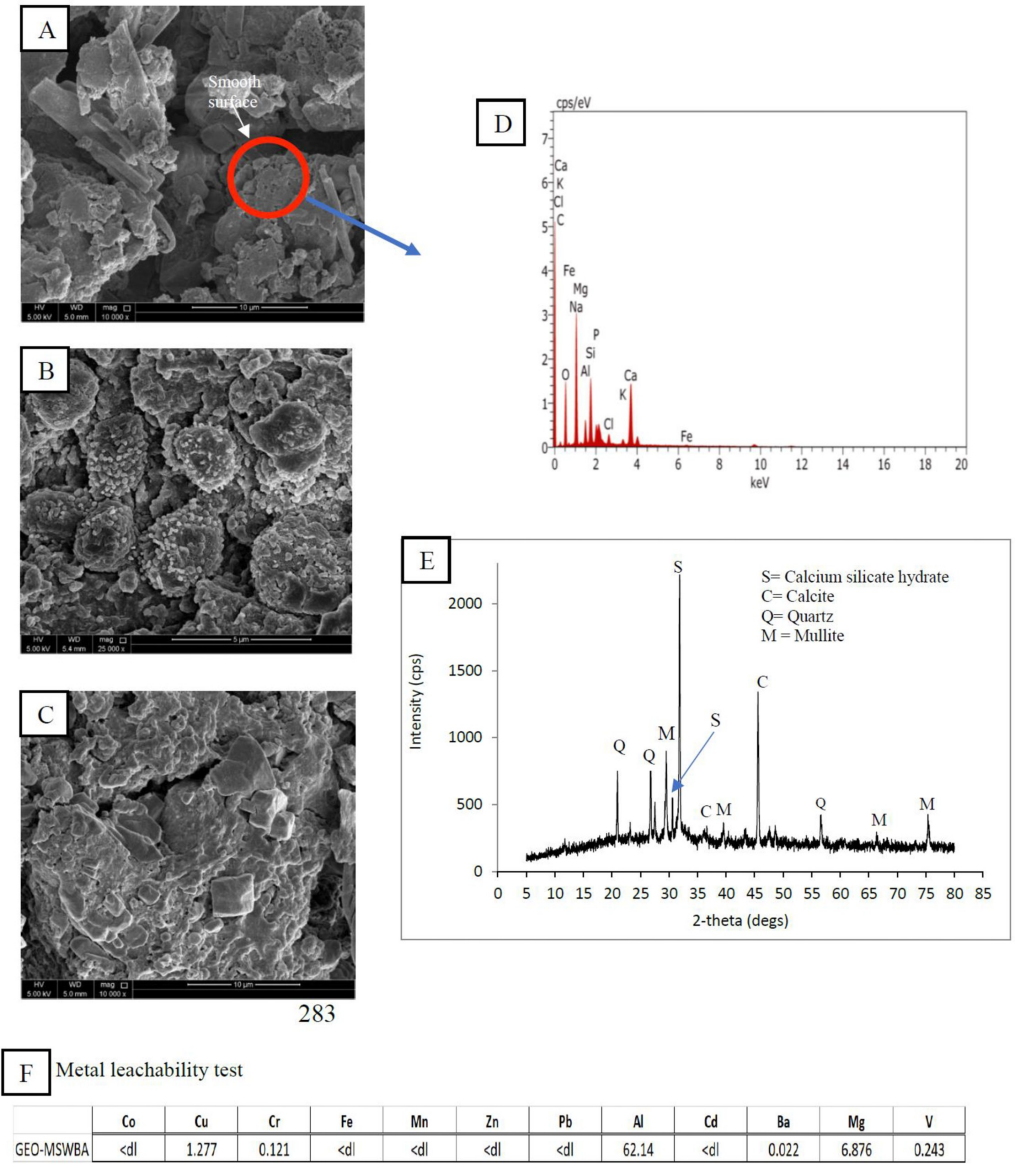
SEM images of (A-C) GEO-MSWBA, (D) EDX images, (E) XRD images, (F) Metal leachability test.

**Fig 2:**
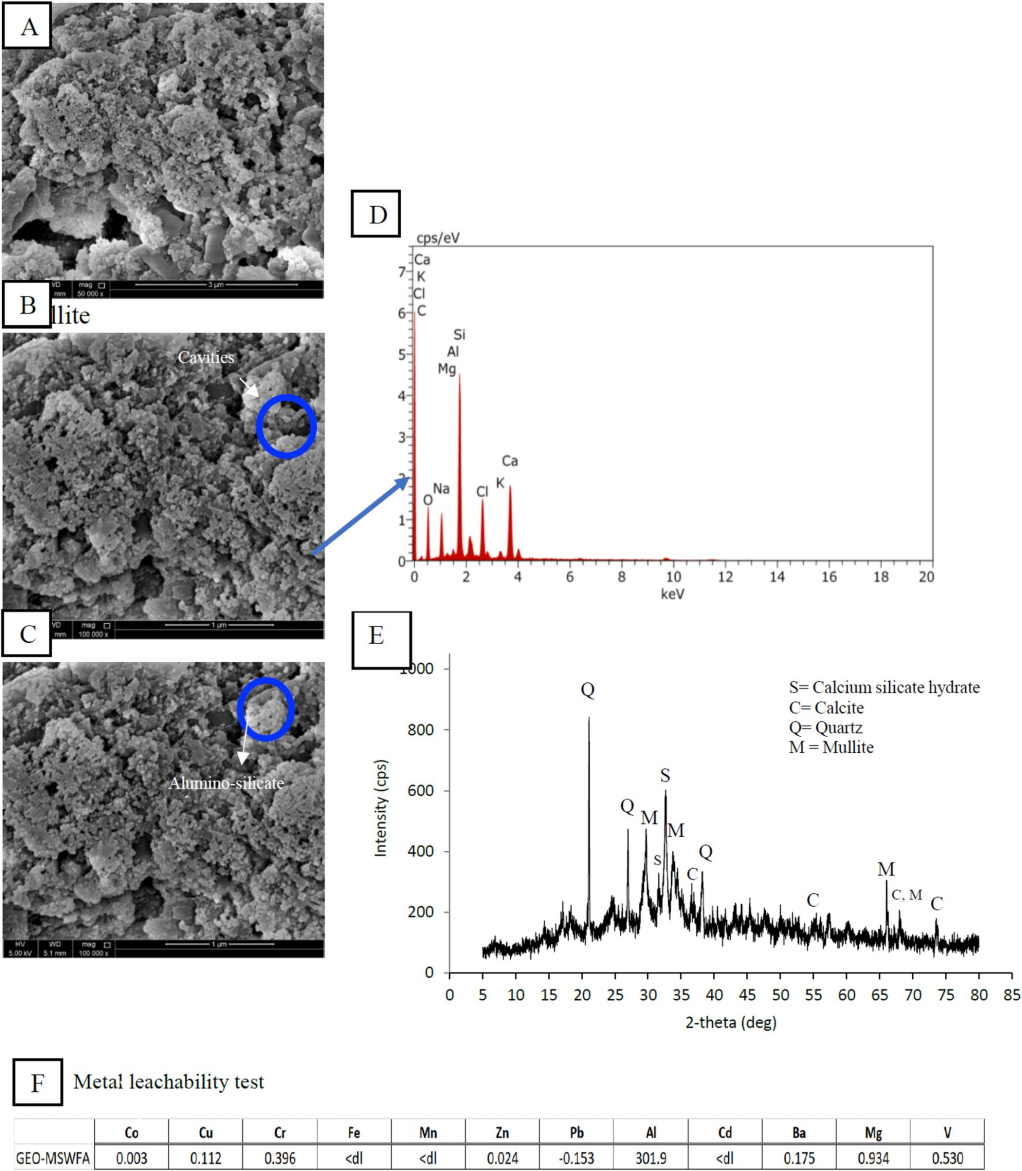
SEM images of (A-C) GEO-MSWFA, (D) EDX images, (E) XRD images, (F) Metal leachability test.

**Scheme 1:**
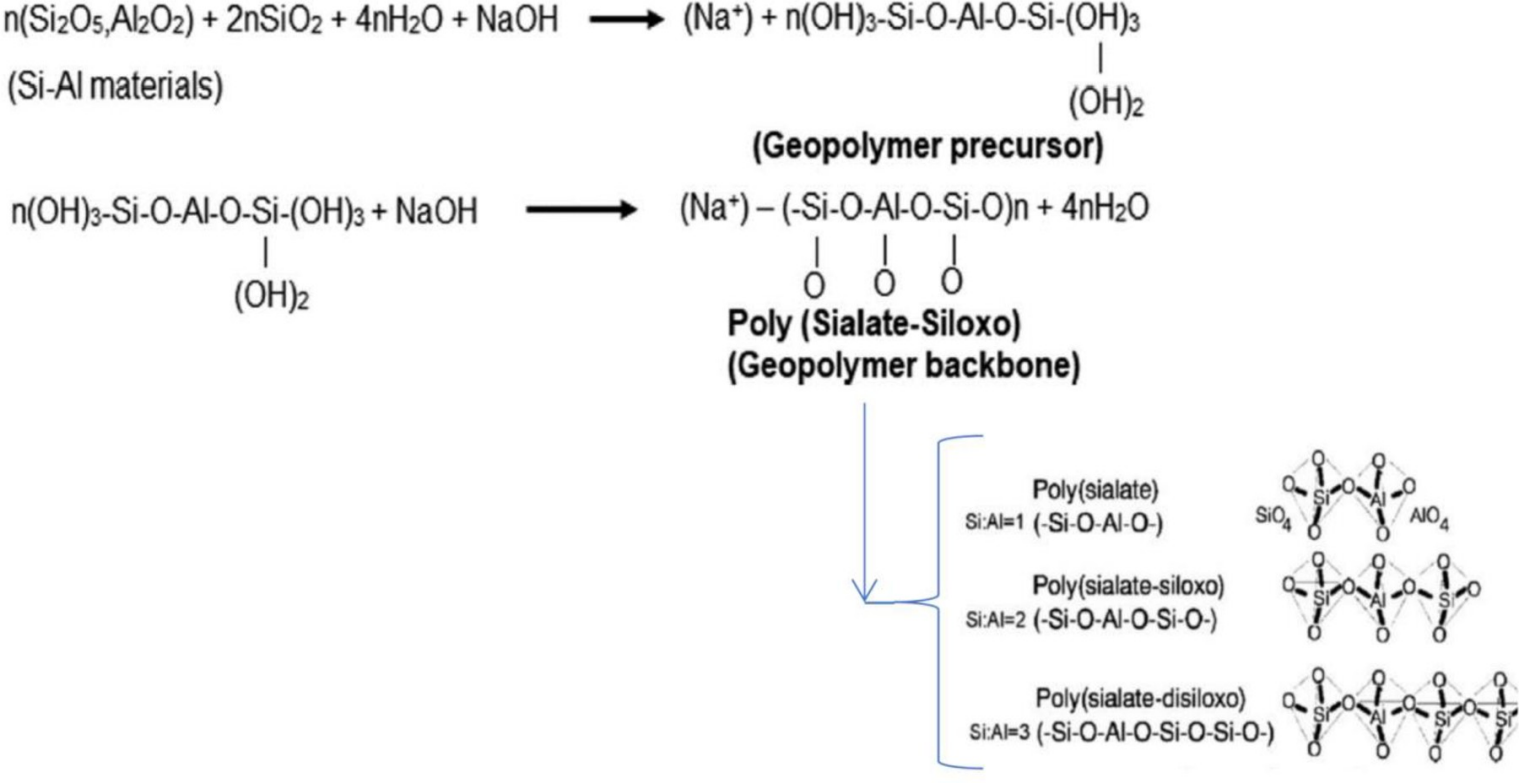
Schematic representation of geopolymerization [37, 38].

Fig 1 shows the morphology and structural attributes of the GEO-MSWBA and the GEO-MSWFA. In both geopolymers, the main characteristics were their spherical shape formation and high porosity. The activation with NaOH led to increased pore density on both GEOs [45]. It is evident that the GEO-MSWBA (Fig 1A to 1C) consisted of a uniform granular and a spongy-gel like structure. Additionally, in Fig 1C, it is apparent that in some places the structure formation was much flatter and consisted of larger spherical shape liked structure. This structure can be described as an intact structure that is bonded together with other small spheres in a dense continuous gel-like matrix. This observation suggested that not all MSW-BA were successfully reacted in the activator solution [46]. The GEO-MSWFA on the other hand consisted of structures, which were denser and packed together (Fig 2A–2C). Furthermore, the tiny structures were more apparent on the GEO-MSWFA, which indicates the geopolymerization of the GEO-MSWFA was comparatively more successful. The different microstructure can perhaps be due to the difference in the characteristic of both MSW-BA and MSW-FA. This tiny structure formation enhanced the removal of MB and played a significant role in the adsorption of MB onto the GEO-MSWBA and GEO-MSWFA. The ashes were cracked under alkaline solution, which caused the active Al and Si to dissolve and form a gel-like matrix structure of sodium aluminosilicate [47]. This sponge-like structure confirmed the successful geopolymerization of the MSW-BA and MSW-FA. As mentioned earlier, the formation of a GEO goes through a continuous process of oligomerization, polymerization, and nucleation [38]. The oligomer framework is closely connected by the covalent oxygens, which specifies to poly(sialates), ply(sialate-siloxo) and poly(sialate-disioxo) as shown in Scheme 1 [48]. It can also be assumed that the metals are trapped in this network structure, which prevents the leaching of metals into the aqueous solution consequently, preventing the metal ions and MB ions to compete for active binding sites on the adsorbent. Here, the metal leachability test was carried out for both geopolymers, and the results are shown in Figs 1F and 2F.

It was noticed that the surface of the MSW-BA and the MSW-FA after geopolymerization became rougher, which indicated the decomposition of clusters. The irregular shape in both GEO-MSWBA and GEO-MSWFA can be related to the amorphous composition of material. The hydrous metal oxide formation changed the material surface further by making it coarser, non-homogenous, with cavities and protuberance. The more porous structure indeed facilitated the adsorption phenomenon for liquid and solid phases. The random arrangement of the structure further helped the adsorption of MB by preventing the escape of ions between the particles by creating a more network-like structure. Similar results were observed in [47, 49]. Chindaprasirt et al. [43] mentioned in their study, that geopolymer prepare by bottom ash was more porous in contrast with the one prepared with fly ash and the particles had more porous like structure in their cavities and irregular in shape. Furthermore, the cavities formed were mostly gels were the result of long chains of silicates and aluminates by the sodium ions. This characteristic along with being porous can be an important factor in the adsorption of metals as the ions can be trapped easily. On the other hand, the elemental composition of the GEO-MSWBA and the GEO-MSWFA and the EDX spectra are shown in Figs 1D and 2D. The main elemental composition of the GEO-MSWBA was C, O Na, Mg, Al, Cl, and Si. While in the GEO-MSWFA, the presence of C, O, Na, Fe, Al, and Si were observed. The presence of calcium can perhaps be from the source material. It is interesting to point out both ashes had different ratios of Si/Al. Chindaprasirt et al. [43] studied the characteristic of the bottom and fly ashes geopolymer and found Si and Al to be the major elements while Na and Ca were present in less percentage. Furthermore, it was suggested that differences in Si/Al ratio in the ashes were due to the leaching of alumina. While, Na, Al, and Si were expected to be present due to the interaction of ashes with sodium silicate [50].

On the other hand, the diffractrograms of the GEO-MSWBA and the GEO-MSWFA were also studied as shown in Figs 1E and 2E, respectively. By observing the intensity and double degree theta (2*θ*), various chemical compositions were observed. The XRD allowed observation of the microstructural transformations that occur after the alkaline activation. In both Figs 1E and 2E, it is possible to observe the decrease of the crystalline peaks as well as the variation of the height and extension of the amorphous halo in the 2θ = 12–40° region. A large hump between 15° and 35° (2θ) was also observed which indicates the existence of an amorphous phase describing the amorphous and crystalline nature of the material. A broad hump between 23–37° theta confirmed the presence of the glassy matrix [51] as well as the crystalline phases of calcite (CaCO_3_) and quartz (SiO_2_). While the peak at 32.5° indicated the presence of calcium silicate hydrate phase in both geopolymers [52]. Furthermore, the peak at 2θ = 25° and 35° can be attributed to silicon oxide. Maleki et al. [49] studied geopolymer characterization and observed similar peaks in addition to cubic structures of magnetite iron oxide nanoparticles. While on the other hand, Wang et al. [46] fly ash geopolymer and found a hump between 25–40°, which were attributed mainly to mullite (Al_6_Si_12_O_13_) and quartz. The intensity of the reflection peaks of calcite is higher in the GEO-MSWBA. This could be related to the presence of higher calcium hydroxide content surrounding calcium silicate hydrate due to the higher CaO content.

#### 3.1.2 Surface area and pore size distribution analysis

The surface area can affect the adsorption rate as well as the uptake capacity. The BET surface areas of the GEO-MSWBA and the GEO-MSWFA were 32.78 m^2^/g and 4.5 m^2^/g, respectively. The high surface area corresponds to perhaps an improvement in surface area of the GEO-MSWBA to adsorb MB more effectively. Ge et al. [53] prepared a green geopolymer and found its surface area 16.2 m^2^/g. While Tang et al. [54] stated metakaolin based geopolymer with surface area 53.95 m^2^/g.

While the total pore volume was also observed to be higher for the GEO-MSWBA which was 0.14 cm^3^/g, and 0.03 cm^3^/g for the GEO-MSWFA. The pore size of the GEO-MSWBA was 64.8 nm while for the GEO-MSWFA was 26.7 nm. When the values were compared with other prepared geopolymers, for instance, Liu et al., 2016, the pore volume and the pore size of the prepared geopolymer were 0.070 cm^3^/g and 19.62 nm, respectively. Similarly, Lee et al. [55] found the pore volume of 0.26 cm^3^/g and pore size to be 8.98 nm. Kara et al. [50] prepared metakaolin based geopolymer and reported the surface area to be 39.24 m^2^/g. Furthermore, Rasaki et al. [19] in their review mentioned fly ash-based geopolymer surface area to be 16.2 m^2^/g, and pore size was 11.5 nm.

#### 3.1.3 FTIR spectra

**Fig 3:**
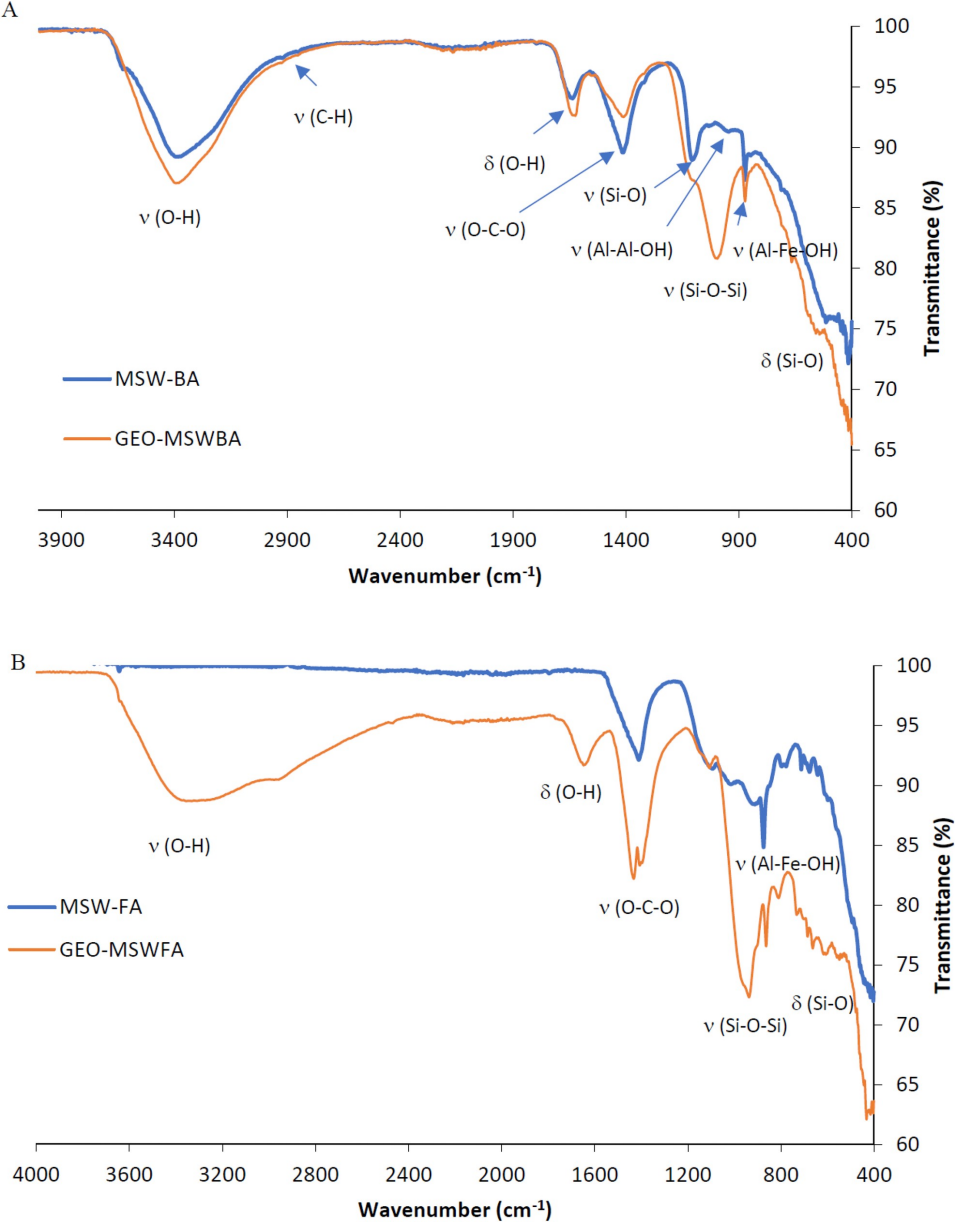
FTIR spectra of A. MSW-BA and GEO-MSWBA and B. MSW-FA and GEO-MSWFA.

Fig 3A shows the spectra of the MSW-BA and the prepared GEO-MSWBA. In the MSW-BA, a bending vibration was observed at 875 cm^-1^, which indicated the presence of Al-Fe-OH while the bending at 975 cm^-1^ indicated the vibration of Al-Al-OH. The vibration at 1115 cm^-1^ indicates the presence of Si-O. Another stretch can also be observed at 1138 cm^-1^, which shows C-O presence. The peak observed between 2900–2800 cm^-1^ shows the C-H aldehydic functional group and the peak at 3381 cm^-1^ indicates the presence of the O-H bond. In contrast, the spectrum of the GEO-MSWBA indicates the presence of stretching vibration of the aldehyde functional group between 2800 cm^-1^ and 2900 cm^-1^, furthermore, the absorption band corresponds to the stretching vibration of aliphatic groups C-H. The presence of a peak at ~1460 cm^-1^ signifies the presence of sodium carbonate [56]. The significant peak between 1650–1600 cm^-1^ is located for O-H bending [43]. While Fig 3B shows the spectra of MSW-FA and the prepared GEO-MSWFA. The peak at 800 cm^-1^ attributes to the presence of Si-O bond while the peak at 1000 cm^-1^ further assures the presence of silica. The peak at 1500 cm^-1^ indicates the C-O bond. 2940 and 2842 cm^-1^ indicate the presence of -CH_3_ asymmetric and -CH_2_ symmetric stretching vibration respectively. In contrast, the GEO-MSWFA spectrum shows a stretching and deformation vibration at 1640 cm^-1^ of OH [46]. Furthermore, at 1018 cm^-1^ the stretching vibration owes it to the asymmetric bond of Al-O and Si-O [57]. Si-O-Si bond was also apparent at 747 cm^-1^ [58]. The stretching vibration at 1200–950 cm^-1^ indicates the presence of Si-O-Si, which indicates the success of the geopolymerization of both ashes. Furthermore, the absorption band at 2800 and 2900 cm^-1^ correspond to the stretching vibration of aliphatic C-H. While the peak at around 1400 cm^-1^ is related to the presence of carbonate. 1410 cm^-1^ in the GEO-MSWFA was due to O-C-O stretching vibration which indicates the presence of sodium carbonate [59]. A similar trend was also noticed in a study compiled by Kumar et al. [60] who observed a peak located at ∼980 cm^-1^ associated with asymmetric Al-O-Al/Si-O-Si stretching. While Barbosa et al. [56] mentioned that the band at 1460 cm^-1^ represents the sodium carbonate resulting from the carbonation. It is important to highlight that in both spectra the bands at 984 cm^-1^ and 677 cm^-1^ corresponded to the aluminosilicate tetrahedral asymmetric and symmetric stretch vibration, respectively [61]. The presence of these peaks indicates the success of the geopolymerization of the MSW-BA and MSW-FA to the GEO-MSWBA and the GEO-MSWFA, respectively.

#### 3.1.4 Effect of pH

**Fig 4:**
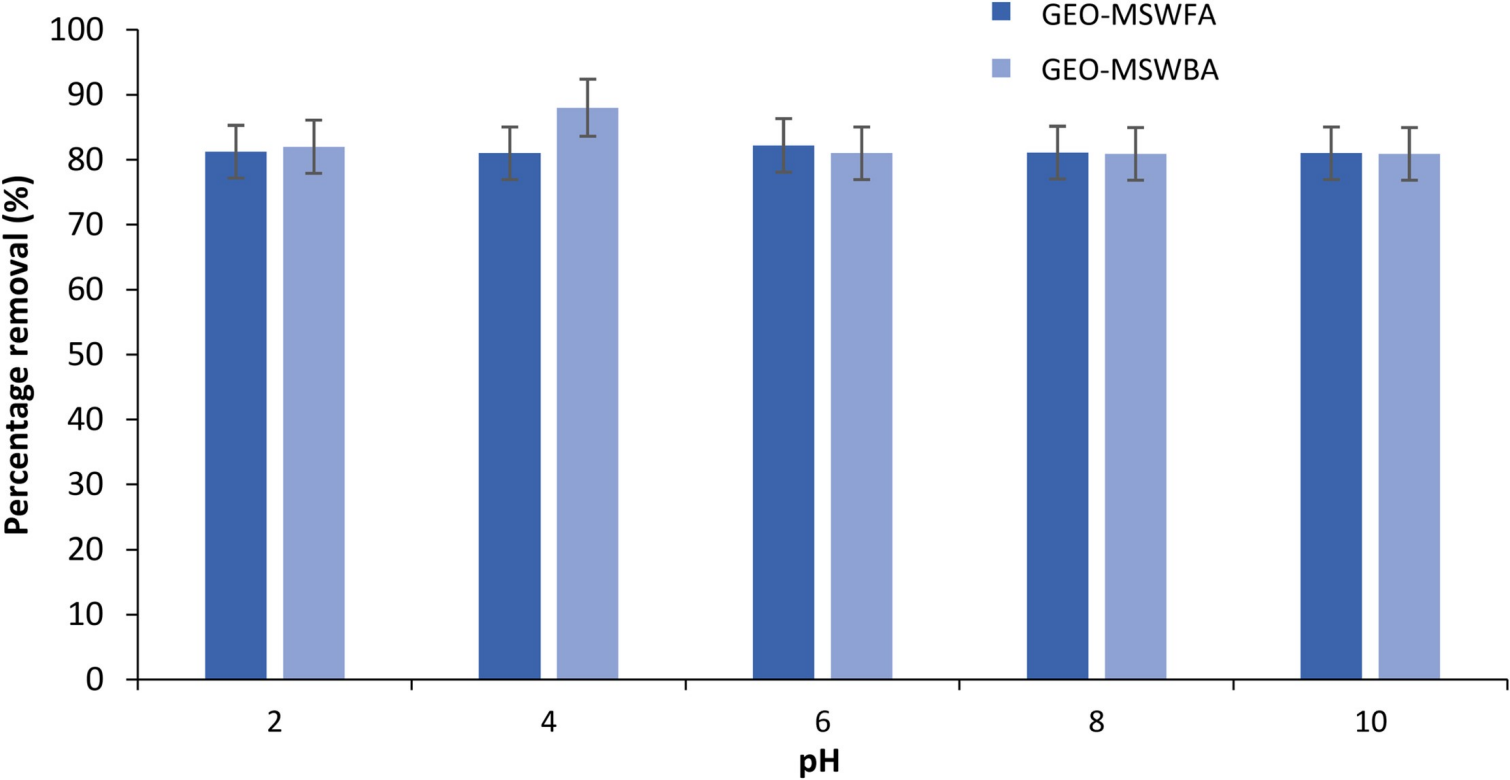
Effect of different pH values on the removal efficiency of MB using the GEO-MSWBA and the GEO-MSWFA.

One of the important parameters in the sorption process is the effect of pH. Changes in the pH solution impacts on the MB adsorption onto the adsorbent. Fig 4 displays the effect of the pH on the MB adsorption capacity onto the GEO-MSWBA and the GEO-MSWFA. Overall, a little decrease in removal percentage was observed as pH values were changed [62]. The pH_solution_ of the prepared geopolymers after 24 h of stirring was 10.01, indicating a strong basicity of the prepared geopolymers. The geopolymer reaction caused a negative solid surface charge [63]. The preference of a low pH perhaps can be explained as at low pH, more positive charges exist on the adsorbent surface, which causes hydroxyl group adsorption. Comparatively, the GEO-MSWBA showed a high MB adsorption at lower pH (88%), which indicates that the zero point of charge occurs at a pH lower than 6. As the pH was increased, lower removal efficiency was observed. This is due to the presence of fewer cations present in the solution to be adsorbed onto the negatively charged geopolymer. Furthermore, it is also possible, that at high pH, the hydroxide ions prevent MB adsorption onto the geopolymers, which ultimately causes a decrease in MB removal. The decrease was also due to the adsorbate and adsorbent ionization, which might have led to the repulsive force between them.

#### 3.1.5 Effect of initial MB concentration

**Fig 5:**
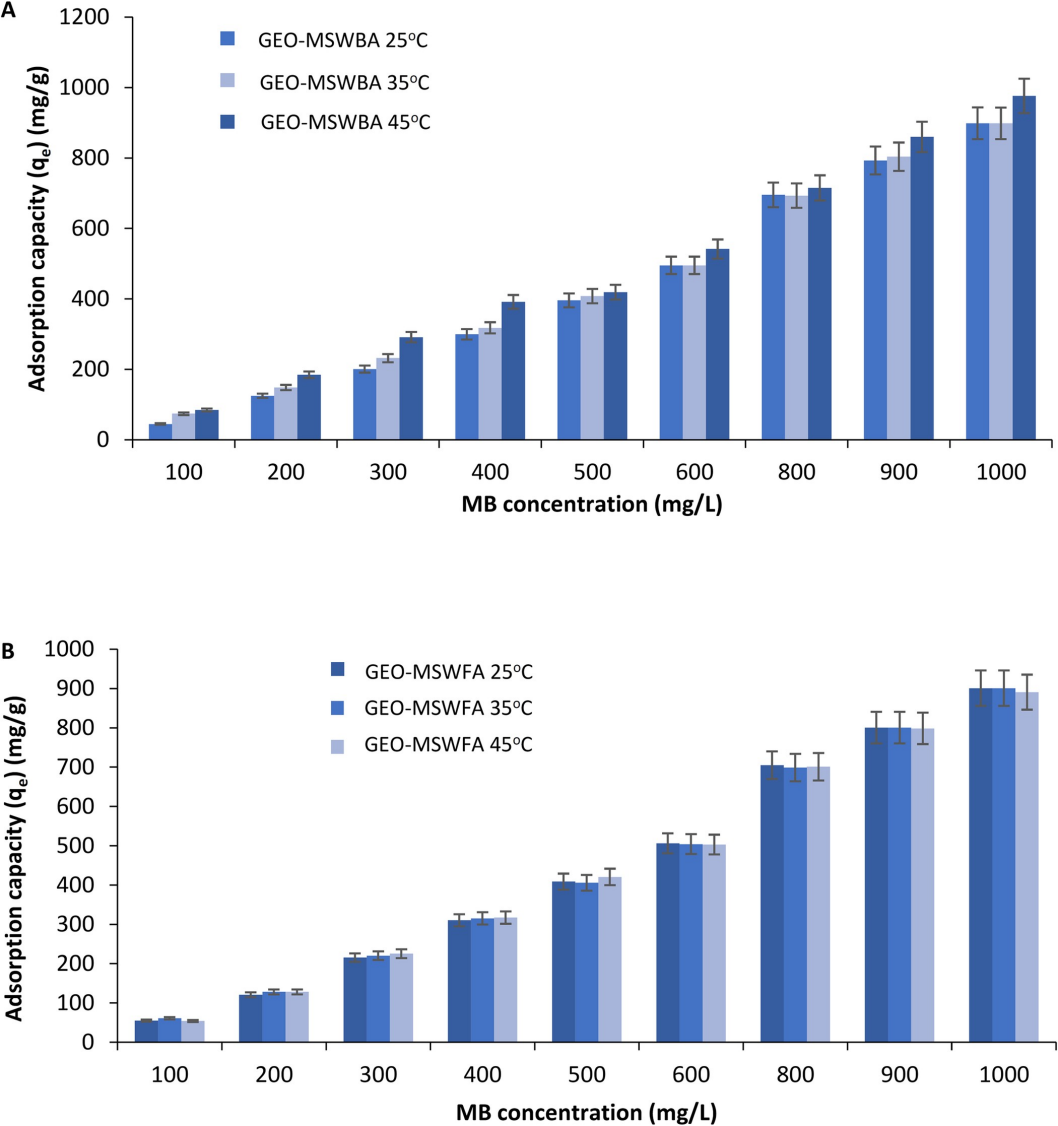
Removal percentage of MB by A. GEO-MSWBA and B. GEO-MSWFA.

Fig 5 shows the effect of initial MB concentration on the removal efficiency using the GEO-MSWBA and the GEO-MSWFA. It is evident that as the concentration of MB increased the amount of MB adsorbed on the adsorbent also increased. For instance, the adsorption capacity (q_e_) was 55.04 mg/g at 100 mg/L while increased to 190.51 mg/g and 800.87 mg/g at 200 mg/L and 900 mg/L, respectively. On the other hand, a similar trend was followed for the GEO-MSWFA, at 100 mg/L was 75.03 mg/g, which further increased to 199.51 mg/g at 200 mg/L. While by 800 mg/L the amount increased to 781.90 mg/g. It was concluded as the concentration of MB increased the amount adsorbed on the GEOs increased. This was due to the sufficient available pores on the adsorbents. It can be said that MB acted as a driving force to prevail in the resistance to the mass transfer of MB between the MB aqueous solution and the adsorbent. For both prepared geopolymers, the adsorption capacity did not increase sufficiently after 800 mg/L, this is probably because the geopolymer reached the maximum adsorption capacity. A study by Maleki et al. [27] also concluded that the increase in MB concentration increased the adsorption rate. A similar trend was observed by Sharifpour et al. [1] who investigated the adsorption of malachite green from aqueous sample byes copper sulfide. Yagub et al. [4] also studied the adsorption of MB on to pine leaves and found similar results.

To validate the data of the effect of pH, temperature, and the initial concentration on the adsorption process, two way ANOVA was performed in this study. The probability value (p-value) can be defined as a significant level, which leads to the null hypothesis rejection. The ratio between the mean square effect, errors, and the p-value is determined using Fischer Variance (F) [64]. Accordingly, if the p-value is less than 0.05 it signifies that the data is significantly different between the main group. According to the data tabulated in Table 1, the p-values for both GEO-MSWBA and GEO-MSWFA for the pH parameter were less than 0.05, indicating the samples are significantly different. It can be observed that overall the p-values were less than 0.05, while F-values were greater than F critic, this signified that the adsorption of MB on the GEO-MSWBA and the GEO-MSWFA was significantly affected by the initial concentration of MB and temperature.

**Table 1:** Analysis of variance for the effect of temperature, pH and initial concentration of MB adsorption onto the GEO-MSWBA and GEO-MSWFA.

Condition	p-value	F-value	F-crit
Temperature GEO-MSWBA	0.00	6.13×10^8^	2.03
pH GEO-MSWBA	0.01	6.20	3.89
Initial concentration GEO-MSWBA	0.23	1.41	2.30
Temperature GEO-MSWFA	0.00	6.55×10^4^	2.04
pH GEO-MSWFA	0.02	5.82	3.89
Initial concentration GEO-MSWFA	0.02	4.58	3.18

### 3.2. Adsorption isotherms of MB onto the GEO-MSWBA and GEO-MSWFA

The Langmuir adsorption isotherm is one of the common isotherm that is used to describe the equilibrium between an adsorbent and adsorbate. In this model, the adsorbent surface is assumed homogenous and there is no interaction between the molecules adsorbed and the adsorbed surface [65]. On the other hand, the Freundlich isotherm model assumes that the adsorption is occurring on a multilayer adsorption. The heat and affinities do not necessarily have to be uniformly distributed to the heterogeneous surface [66]. Dubinin Radushkevich is used to describe the adsorption with the distribution of Gaussian energy on the heterogeneous surface. Lastly, the Temkin model takes into consideration the interaction between the adsorbent and adsorbate ignoring the low concentration value. As the surface coverage increase, this model assumes the adsorption heat as a function of temperature for all the molecules existing in the layer to decline linearly rather than logarithmically [67]. Table 2 summarizes the four isotherm equations and the parameters that were used in this study.

**Table 2:** The studied isotherm models linear equation and plots.

Adsorption model	Equation	Parameter	Plot
Langmuir isotherm [65]	$\frac{C_{e}}{q_{e}}=\frac{1}{Q^{0}K_{L}}+\frac{1}{Q^{0}}C_{e}$	*qe* is the equilibrium adsorbent-phase concentration of adsorbate (mg/L) while, *Ce* represents the equilibrium aqueous-phase concentration of adsorbate (mg/L). *Q*^0^ is the maximum monolayer adsorption capacity (mg/g), and *K*_*L*_ is the constant related to the free adsorption energy and the reciprocal of the concentration at which half-saturation of the adsorbent is reached.	$\frac{Ce}{q_{e}}$ vs. *Ce*
Freundlich isotherm [68]	$logq_{e}=logK_{f}+\frac{1}{n}logC_{e}$	qe is the amount of adsorbate in the adsorbent at equilibrium (mg/g), Freundlich adsorption constant is denoted by *K*_*f*_ (mg/g)(L/g)n, and *C*_*e*_ is the equilibrium constant (mg/L). while the intensity of the adsorption process is determined by n	log(*qe*) vs. log (*Ce*)
Dubinin-Radushkevich isotherm [69]	*lnq*_*e*_ = *q*_*s*_−*K*_*DR*_ε^2^	K_DR_ is the Dubinin Radushkevich isotherm constant (mol^2^/kJ^2^), *qs* is the theoretical isotherm saturation capacity (mg/g) and *q*_*e*_ is the amount of adsorbate in the adsorbent at equilibrium (mg/g), ε = RT ln[1+1/C_e_].	*ln(qe)* vs. ε^2^
Temkin isotherm [70]	$q_{e}=\frac{RT}{B_{T}}\ln A_{T}+(\frac{RT}{B_{T}})\ln C_{e}$	R is the universal gas constant (8.314 J/mol K), T is the temperature in Kelvin (K), *B*_*T*_ is the Temkin isotherm constant which is related to sorption heat (J/mol), *qe* is the amount of adsorbate in the adsorbent at equilibrium (mg/g), AT is the Temkin isotherm equilibrium binding constant (L/g) and *Ce* is the equilibrium concentration (mg/L).	*ln(qe)* vs. *lnC*_*e*_

Adsorption isotherm illustrates the interaction between adsorbate and the adsorbent occurring at either maximum adsorption capacity or equilibrium [71]. Isotherms are also used to determine the efficiency of the adsorbent. The results of the MB adsorption onto the GEO-MSWBA and GEO-MSWFA using the four isotherms are tabulated in Table 4. For the Langmuir isotherm, the correlation coefficient (R^2^) was close to 1; (0.94–0.99) for the GEO-MSWBA, and (0.92–0.98) for the GEO-MSWFA. This indicated that the MB adsorption onto the GEO-MSWBA and the GEO-MSWFA could be best explained by the Langmuir isotherm. The *R*_*L*_, which represents the separation factor was found to be between 0 < *R*_*L*_ < 1 for both adsorbents, showed that the process was energetically favorable. The *K*_*L*_ constant, which refers to the affinity between the adsorbate and adsorbent, suggests that there was comparatively stronger binding at 45°C for the GEO-MSWBA, while for the GEO-MSWFA at 25°C showed a high affinity. Furthermore, the *Q*^0^ which denotes the maximum adsorption capacity showed that as the temperature increased the maximum capacity decreased for the GEO-MSWBA; 666.7 mg/g at 25°C while 476.2 mg/g at 45°C. However, for the GEO-MSWFA, the maximum capacity increased at 35°C, from 434.7 mg/g at 25°C to 769.3 mg/g at 35°C.

The Freundlich isotherm assumes that the uptake of MB ion took place on a heterogeneous surface, and the distribution of heat was non-uniform over the surface. From Table 3, the K_f_ value showed an overall increase as the temperature increased 3.42 (mg/g)(L/g)n and 10.31 (mg/g)(L/g)n at 35 and 45°C, respectively, which indicated that as the temperature increased the adsorption capacity also increased. While for the GEO-MSWFA, as temperature increased the K_f_ value decreased, which indicated that the high temperature did not favor the adsorption process. The *n* value explains if the adsorption was chemical adsorption or physical adsorption. Less than 1 indicates a chemical adsorption while more than 1 shows the adsorption was a physical process. For the GEO-MSWBA and the GEO-MSWFA, the reaction seemed to follow a physical adsorption. While the value for 1/*n* displays the reaction was favorable. However, the R^2^ values for the GEO-MSWBA were low, ranging between 0.74–0.52 while the GEO-MSWFA was between 0.88–98. This suggests that the Freundlich model might not be suitable to describe the adsorption process of the GEO-MSWBA.

**Table 3:** Various studies on MB adsorption using industrial waste.

Adsorbent	Adsorbate	Adsorptive capacity (mg/g)	Reference
GEO-MSWBA	Methylene Blue	666.7 at 25°C	Current study
GEO-MSWFA	Methylene Blue	769.2 at 35°C	Current study
Modified Cu_2_O/TiO_2_	Methylene Blue	16.7–20.1	[75]
Zeolite/hydrous from coal fly ash	Methylene Blue	18.94	[76]
Fly ash derived zeolite	Methylene Blue	23.70	[77]
Zeolite synthesized from coal ash	Methylene Blue	37.04–50.51	[78]
Natural Zeolite	Methylene Blue	16.37	[79]
Fly ash	Methylene Blue	5.57	[80]
Fly ash	Methylene Blue	7.07	[81]
Fly ash derived zeolite	Methylene Blue	12.64	[82]

The Temkin isotherm equilibrium binding constant (A_T_) showed an increase and decrease as the temperature increases. For instance, the A_T_ value was 0.02 L/g at 25°C and changed to 23.31 L/g at 35°C and 0.11 L/g at 45°C for GEO-MSWBA, while for the GEO-MSWFA, the A_T_ value was 0.05 L/g at 25°C and changed to 33.89 L/g at 35°C and then dropped to 21.57 L/g at 45°C. The heat adsorption constant (B_T_) showed that the heat adsorption was high at 25°C (114.4 J/mol) and 35°C (96.92 J/mol) for the GEO-MSWBA while for the GEO-MSWFA, it was high at 35°C (174.2 J/mol) and 45°C (146.7 J/mol). Lastly, for the Dubinin-Radushkevich, R^2^ ranged between 0.27 to 0.76 for 35 and 45°C, which suggested that the model was not suitable to describe the adsorption of MB onto the GEO-MSWBA and the GEO-MSWFA.

From the data, it can be said that the GEO-MSWBA and the GEO-MSWFA can be best explained by the Langmuir model, which assumes that the adsorption was carried out on a homogenous site and the occurrence of the reaction was by monolayer formation without any internal interaction among the adsorbed ions. The maximum adsorption capacity was 666.7 mg/g for the GEO-MSWBA while 769.2 mg/g for the GEO-MSWFA. It can be concluded that GEO-MSWFA was a better adsorbent in contrast with the GEO-MSWBA. This can perhaps be due to the difference in the structural formation, as mentioned earlier the SEM images of the GEO-MSWBA showed a much longer and flatter surface while the GEO-MSWFA consisted of more packed cavities like structure, which enhanced the porosity of the adsorbent and aided in capturing and binding the MB ions on to the surface. This coincides with similar findings [72, 73]. Alouani et al. [74] studied the adsorption capacity of activated coal fly ash. It was found that the Langmuir model was the best suited to describe the adsorption process with the maximum monolayer adsorption capacity of 37.08 mg/g. Khan et al. [73] prepared an acid-based geopolymer the adsorption, and it was best explained using the Langmuir isotherm with maximum adsorption capacity reaching 3.01 mg/g. When comparing MSW geopolymer with other prepared geopolymers, it can be said that MSW-geopolymer exhibited a better adsorption capacity for MB.

Table 3 illustrates a comparison of the adsorbent capacity of the current study in contrast with other adsorbents that were reported in various literature to remove MB [75–82]. The table shows that the GEO-MSWBA and the GEO-MSWFA were superior to most of the adsorbents as they showed very high adsorption capacities for MB. Thus, it can be concluded that the prepared GEO-MSWBA and GEO-MSWFA were excellent adsorbents that able to adsorb up to 769.2 mg/g MB from aqueous solution and also featuring notable removal percentages.

The K_DR_ value of the Dubinin-Radushkevich model could be a useful magnitude for the interpretation of the MB adsorption mechanisms onto the GEO-MSWBA and the GEO-MSWFA. The K_DR_ in the Dubinin–Radushkevich isotherm represents the mean free energy (E) of adsorption per mole of the adsorbate (kJ/mol). The E_D_ can be obtained from the Eq 3:

$$E_{D}=\frac{1}{\sqrt{2K_{DR}}}$$ (3)

The physical adsorption mechanism will be dominant when the E_D_ value is less than 8 kJ/mol. However, the ion exchange mechanism will be governed when the E_D_ value is between 8 and 16 kJ/mol. For the value of E_D_ greater than16 kJ/mol, the particle diffusion phenomenon will be dominant.

To distinguish between the physical and chemical adsorption of MB ions, the E_D_ (per molecule of adsorbate) from the Dubinin-Radushkevich adsorption model was used in finding the most-stable MB adsorbed onto the surfaces of the GEO-MSWBA and GEO-MSWFA. For example, in the MB adsorbed onto the GEO-MSWFA system, the E_D_ was calculated according to the Eq 4,

$$\Delta E_{D}=[E_{MB\;adsorbed\;on\;GEO-MSWFA}-(E_{GEO-MSWFA}+E_{MB})]$$ (4)

Where E_MB adsorbed on GEO-MSWFA_, E_GEO-MSWFA_ and E_MB_ are total energies of the MB ions adsorbed GEO-MSWFA system, the GEO-MSWFA surface, and single MB ions, respectively.

**Fig 6:**
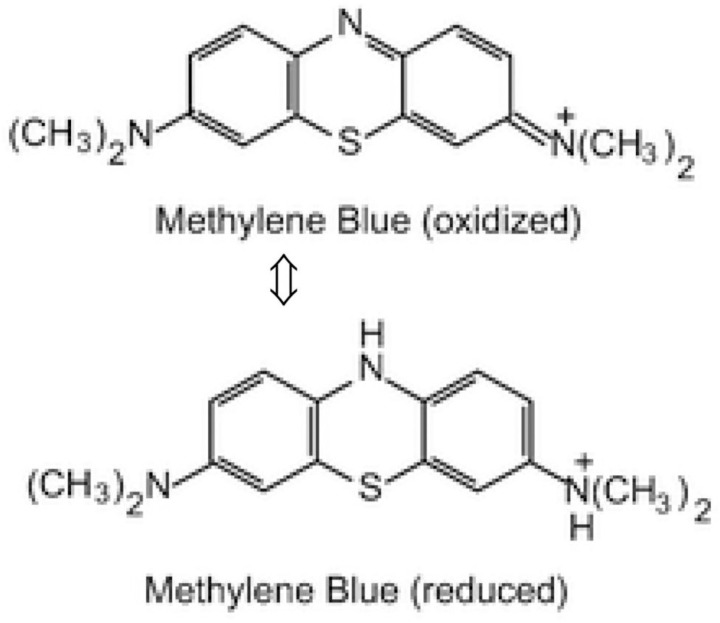
Molecular structure illustrating the reduction and oxidation of MB equilibrium.

Table 3 indicates that the GEO-MSWFA has the lowest free energy (E_D_) of the adsorption per mole of the adsorbate (9.71 kJ mol^-1^ at 25°C); suggesting that the adsorption process is dominated by an ion-exchange mechanism. This variation on E_D_ values for the GEO-MSWFA could be related to the MB adsorption profile onto the surface of the GEO-MSWFA and the MB oxidation and reduction. This also could be explained as once the MB adsorbed on the surface of the GEO-MSWFA may undergo an equilibrium of reduction and oxidation, and the MB configuration could be changed according to the state of MB and the amount of energy required for adsorption (Fig 6).

The Langmuir Q^0^ parameter can be linked to the variation of the energy adsorption; however, the B_T_ value of the Timken equation only allows to access this energy variation. Here, the heat of adsorption varies linearly with the degree of MB covering (θ;*θ* = *f*(*lnC*_0_)) onto the surface of the GEO-MSWFA as shown in Eq 5. The linear decrease could be explained on a uniform surface by interactions between MB molecules adsorbed. On a non-uniform surface, this effect can be superimposed on those due to the heterogeneity of the surface. Table 4 shows that the B_T_ values are positive for the GEO-MSWBA and the GEO-MSWFA, which means that the adsorption reaction is exothermic (substitution of one or more water molecules by an organic molecule).

$$\theta =\frac{RT}{\Delta B_{T}}.lnA_{T}C$$ (5)

A_T_ is the adsorption-adsorbent Temkin constant (L/mol), ΔB_T_ is the variation of the adsorption energy (kJ/mol) of Temkin.

**Table 4:** Adsorption isotherm parameters of the MB adsorption onto the GEO-MSWBA and the GEO-MSWFA.

**GEO-MSWBA**								
**Temp.**°C	**Langmuir**				**Freundlich**			
	**Q**^**0**^ **(mg/g)**	**K**_**L**_ **(L/mg)**	**R**^**2**^	**K**_**f**_ **((mg/g)(L/g)**^**n**^**)**	***n***	**1/*n***	**R**^**2**^
25	666.7	0.001	0.9980	3.42	1.38	0.72	0.7400
35	454.5	0.003	0.9497	8.08	1.69	0.59	0.5200
45	476.2	0.004	0.9513	10.31	1.79	0.55	0.9300
**Temp.**	**Temkin**				**Dubinin-Radushkevich**			
	**A**_**T**_ **(L/g)**	**B**_**T**_ **(J/mol)**	**B**_**T**_	**R**^**2**^	**qs**	**K**_**DR**_ **(mol**^**2**^**/kJ**^**2**^**)**	**R**^**2**^	**ED (kJ/mol)**
25	0.02	114.4	19.84	0.7900	266.4	0.0010	0.56	22.4
35	23.3	96.72	26.47	0.9400	275.9	0.0011	0.75	21.3
45	0.11	80.54	32.82	0.9000	595.0	0.0010	0.70	22.4
**Temp**°C	**Langmuir**				**Freundlich**			
	**Q**^**0**^ **(mg/g)**	**K**_**L**_ **(L/mg)**	**R**^**2**^	**K**_**f**_ **((mg/g)(L/g)**^**n**^**)**	***n***	**1/*n***	**R**^**2**^
25	434.7	0.004	0.9200	28.44	2.40	0.42	0.8700
35	769.2	0.003	0.9800	10.73	1.61	0.62	0.9800
45	714.3	0.004	0.9700	9.115	1.52	0.66	0.9700
**Temp**	**Temkin**				**Dubinin-Radushkevich**			
	**A**_**T**_ **(L/g)**	**B**_**T**_ **(J/mol)**	**B**_**T**_	**R**^**2**^	**qs**	**K**_**DR**_ **(mol**^**2**^**/kJ**^**2**^**)**	**R**^**2**^	**ED (kJ/mol)**
25	0.05	90.38	25.10	0.8500	0.99	0.0053	0.9500	9.71
35	33.9	174.2	14.70	0.9800	399.4	0.0011	0.8600	21.3
45	21.6	146.7	18.02	0.9600	444.0	0.0036	0.8700	11.8

### 3.3. Thermodynamics studies

Thermodynamic parameters, i.e., enthalpy (Δ*H*^*o*^), Gibbs free energy (Δ*G*^*o*^), and entropy (Δ*S*^*o*^) were determined by using the effect of temperature and van't Hoff equation. The relationship between the adsorption coefficient *K*_*L*_ from the Langmuir adsorption isotherm and the temperature is expressed as Eqs 6 and 7 [81].

$$\Delta G^{o}=\Delta H^{o}-T\Delta S^{o}$$ (6)

$$\Delta G^{o}=-RTLnK_{L}$$ (7)

Where Δ*H*^*o*^ is enthalpy change (kJ/mol), Δ*S*^*o*^ is entropy change (J/mol.K), and Δ*G*^*o*^ is Gibbs free energy change (kJ/mol). *K*_*L*_ is the Langmuir adsorption equilibrium constant (L/mol), *R* is the gas constant (8.314 J/mol.K) and *T* is the absolute temperature (K).

From the data in Table 5, it was found that the MB adsorption onto the GEO-MSWBA and the GEO-MSWFA was spontaneous and feasible as the value of ΔG^*o*^ becomes more negative with an increase in temperature. The decrease in ΔG^*o*^ with an increase in temperature also showed that the adsorption process was endothermic. The positive value of enthalpy (ΔH^*o*^) for the GEO-MSWBA indicated that the process followed an endothermic reaction, in addition, the negative value of ΔS^*o*^ indicated solid/liquid random interaction throughout the adsorption process [83]. While for the GEO-MSWFA, it followed an exothermic reaction. While the positive value of entropy (ΔS^*o*^) for GEO-MSWFA suggested that the MB adsorption onto GEO-MSWFA had a good affinity at the solid-liquid surface.

**Table 5:** Thermodynamics properties of the MB adsorption onto the GEO-MSWBA and GEO-MSWFA.

Temperature (°C)	ΔG° ****(kJ/mol)****	ΔH° ****(kJ/mol)****	ΔS° ****(J/mol.K)****
GEO-MSWBA		21.14	-720.3
25	-78.75		
35	-10.45		
45	-10.48		
GEO-MSWFA			
25	-47.88	-19.11	53.23
35	-11.18		
45	-56.77		

### 3.4. Adsorption mechanism

**Fig 7:**
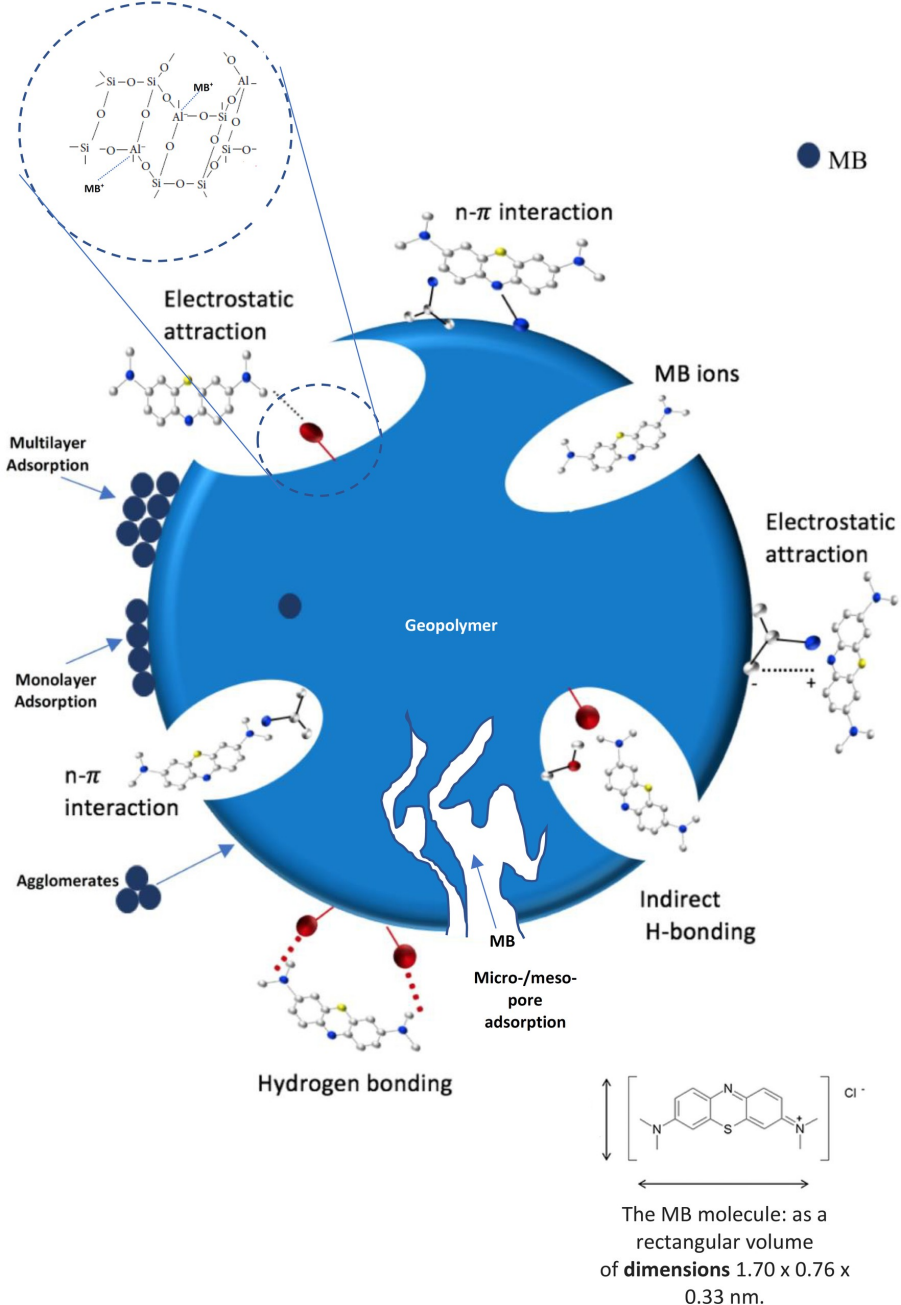
Schematic representation of MB adsorption mechanisms onto the GEO-MSWBA or the GEO-MSWFA.

The adsorption characterizations of MB onto the GEO-MSWBA and GEO-MSWFA would help in understanding the adsorption behavior and mechanisms of MB onto these adsorbents. As observed from the SEM images and the pores size distribution, the prepared GEO-MSWBA and GEO-MSWFA consisted of micropores (the pore size of GEO-MSWBA was 64.8 nm while for GEO-MSWFA was 26.7 nm), which accelerated the mass transfer of MB onto the adsorbate (Fig 7). It can be assumed the metal ions substitution or silico-aluminum substitution caused the adsorbate surface to be negative offering electrostatic interaction between the geopolymer surface and the cationic MB dye. The electrostatic interaction is greatly affected by the molecular structure of the dye due to the steric effect [82]. The structure of MB is thought to be as leaner or lying flat on the surface leading to a simple electrostatic interaction between the surface of the adsorbent and the dye [84]. The presence of metal ions on the surface of a geopolymer such as Al^3+^, Mg^2+^, and K^+^ and the conjugated structure of the MB molecule might have also caused *n*- *π* interaction (Fig 7). Furthermore, the strong polarizing power of Al^3+^ might have led to the dissociation of water and formation of Al-OH indicating the presence of indirect hydrogen bond, while the direct formation of hydrogen bond might between the H of hydroxyl groups and N or S in the MB dye. Fig 7 shows a schematic representation of MB adsorption mechanisms onto the GEO-MSWBA or GEO-MSWFA.

## 4. Conclusion

This work demonstrated a successful preparation of low-cost adsorbents prepared by geopolymerization. In the present study, the GEO-MSWBA and GEO-MSWFA were prepared using MSW-BA and MSW-FA. The presence of alumino-silicate gel demonstrated the success of geopolymerization. Furthermore, the morphology of the GEO-MSWBA and GEO-MSWFA also transformed from smooth to a rough surface consisting of much more dense pores in addition the SEM graphs also showed an increase in specific surface area as well as morphological amendments. The presence of the Al-O-Al/Si-O-Si functional groups that were also found in FTIR further confirmed a successful geopolymerization. The adsorption process in both adsorbents was best described using the Langmuir model. The maximum MB adsorption capacity was 666.7 mg/g (at 25°C) for the GEO-MSWBA while 769.2 mg/g (at 35°C) for the GEO-MSWFA. The thermodynamic functions such as ΔG°, ΔH°, and ΔS° in the adsorption process were also examined. The negative value of ΔS^*o*^ for GEO-MSWBA indicated random state at solid-liquid interference throughout the adsorption process of MB while the positive value of ΔS^*o*^ for GEO-MSWFA suggested that the MB adsorption had a good affinity at the solid-liquid surface. The negative value for ΔG^*o*^ for both adsorbents indicated that the reaction was spontaneous and feasible. The studies also concluded that the reaction followed an exothermic reaction for the GEO-MSWBA and favored low temperature (25 ^o^C). While the GEO-MSWFA followed an endothermic reaction and was more feasible at a slightly higher temperature (35 ^o^C).

The outcome of this study also sheds light on the ability of utilizing local by-products as a highly efficient, potentiated, and inexpensive adsorbents in contrast to other expensive adsorbents offered in the market. It possesses advantages such as high adsorption efficiency, easy separation from the liquid solution, waste minimization, cutback in landfilling practices, and preventing the utilization of virgin raw material. For future studies, the adsorbents shall be further exploited in order to enhance the geopolymer capabilities of effectively removing other cationic dyes from dyeing wastewater or other emerging pollutants and also explore the possibility of utilizing it on a larger scale.
